# Intensive Treatment of Cellulite Based on Physiopathological Principles

**DOI:** 10.1155/2012/834280

**Published:** 2012-05-14

**Authors:** José Maria Pereira de Godoy, Mayra Yara Groggia, Lucilene Ferro Laks, Maria de Fátima Guerreiro de Godoy

**Affiliations:** ^1^Department of Cardiology and Cardiovascular Surgery, Medical School of São Jose do Rio Preto (FAMERP), Avenida Constituição 1306, 15025-120 São Jose do Rio Preto, Brazil; ^2^Medicine School of São Jose do Rio Preto and Godoy Clinic, 15025-120 São Jose do Rio Preto, Brazil

## Abstract

*Aim*. The aim of this paper is to report a novel intensive therapy of gynoid lipodystrophy (cellulite) based on a new hypothesis. *Methods*. Were evaluated in 10 patients with ages ranging between 25 and 59 years (mean 35.6 years) grade IV cellulite identified by clinical evaluation. Before initiating treatment, perimetry was performed at 5 cm intervals along both thighs, at the gluteal fold, 5, 10, and 15 cm above the gluteal fold, at the navel, and 5 cm above the navel. The patients were submitted to a 4-hour daily treatment session that consisted of manual and mechanical lymph drainage and cervical stimulation by the Godoy & Godoy technique adapted for the treatment of cellulite. After 10 sessions over two weeks, the patients were again evaluated. The paired *t*-test was utilized for statistical analysis with an alpha error of 5% (*P* value < 0.05). A reduction was identified at all of the measurement points (paired *t*-test; *P* value < 0.0001). *Results*. The mean reductions varied between 4.0 and 5.7 cm at the measurement points but reductions of more than 10 cm in perimeter were achieved in some patients. *Conclusion*. This technique involving lymphatic system stimulation is efficacious in the treatment of cellulite.

## 1. Introduction

 Gynoid lipodystrophy (cellulite) can be regarded as the most investigated nondisease, because, from the cosmetic viewpoint, most women desire a reduction in the severity of cellulite [1]. There is a topographic alteration particularly in hips, buttocks, thighs, and abdomen, where fat deposit seems to be under the influence of estrogen [2].

Weight loss has been reported to improve the severity of cellulite as surface topography measures diminish; however, in obese individuals skin dimpling does not seem to change appreciably. Histological analysis suggests that fat globules retract out of the dermis with weight loss [3]. Only a limited number of studies on cellulite have been published in the international literature and many of them reach somewhat divergent conclusions. Consequently, it is not yet possible to reconcile the extreme differences in opinion, which have lingered for years concerning the nature of this disorder, including its origin and even the most basic aspects of its histopathological classification [4].

 Although no physiopathological basis for cellulite has been defined, several therapies have been employed [5–9]. In fact, no treatment is completely successful, none are more than mildly and temporarily effective [10].

 The aim of the current study is to report on a novel form of intensive treatment for cellulite based on a new physiopathological concept [11].

## 2. Method

 A novel form of cellulite therapy was evaluated in ten patients with ages ranging between 25 and 59 years (mean 35.6 years). The patients were enrolled in the study by order of arrival in the clinic and on acceptance to participate. The inclusion criterion was the presence of the most advanced grade of cellulite according to the clinical diagnosis. The exclusion criteria were a history of edema, obesity, only moderate or mild cellulite, or any other disease diagnosed at the time of the initial interview. Before initiating the treatment, perimetry was performed at 5 cm intervals along the thighs starting at the gluteal fold and in the abdomen region: 5, 10, and 15 cm below the navel, at the navel, and 5 cm above the navel. Standardized photographs were taken of all participants. The patients were submitted to a 4-hour daily treatment regimen that consisted in manual and mechanical lymph drainage and cervical stimulation utilizing the Godoy and Godoy technique adapted for the treatment of cellulite [12–16]. After 10 sessions (40 hours of treatment) over two weeks, the patients were again clinically evaluated by perimetry and photographs were taken. Each treatment session comprised four hours of continuous mechanical lymph drainage associated to 15 minutes of cervical stimulation and two hours of manual lymph drainage. The apparatus used for mechanical lymph drainage performs 25 to 30 flexion and extension movements of the sole of the foot per minute [12]. The Godoy and Godoy lymph drainage technique uses continuous manual compression along the path of collectors up to the corresponding lymph nodes. The photographs before and after treatment were compared by two examiners to identify changes. The greatest variation of the thigh and the abdomen was considered for each patient. The paired *t*-test was utilized for statistical analysis with an alpha error of 5% (*P*-value < 0.05) being considered acceptable. 

## 3. Results

 Significant reductions were observed for all the measurement points (paired *t*-test: *P* value < 0.0001). The mean of the circumferences 5, 10, and 15 cm above the gluteal fold decreased from 102.9 cm to 97.3 cm giving a statistically significant mean reduction of 5.6 cm (paired *t*-test: *P* value < 0.0001—Table 1). The mean of the reductions of the right thigh 5 and 10 cm below the gluteal fold was 4.9 cm (paired *t*-test: *P* value < 0.0001—Table 2). The mean of the reductions of the left thigh 5 and 10 cm below the gluteal fold was 4.7 cm (paired *t*-test: *P* value < 0.0001—Table 3). The mean reduction at the gluteal fold was 4.2 cm (paired *t*-test: *P* value < 0.0001—Table 4). The mean reduction at the navel was 3.9 cm (paired *t*-test: *P* value < 0.0001—Table 5). The mean reduction 5 cm below the navel was 4.0 cm (paired *t*-test: *P* value < 0.0001—Table 6).

## 4. Discussion

 The current study shows a novel intensive therapy for cellulite based on a new physiopathological concept. This form of treatment has not previously been published in the literature and aims at physiologically stimulating the lymphatic system. In this study, the patients were evaluated after 10 sessions (40 hours), the period in which the greatest variations are observed. In the first days of the treatment, progressive softening of the tissue is seen, and on about the 6th day, reductions in the perimetry are noticed. Thus, at least ten sessions are required to evaluate the technique for any specific patient.

 A minimum of three sessions should be performed per week, although the authors recommend five. This is important as there is a difference in the results if the treatment method is not followed correctly. The patients were evaluated after 10 sessions, but they continued treatment until the cellulite had disappeared completely. It is important to remember that the inclusion criterion of this study was that only severe cases were selected. In these cases, it was expected that more than 10 cm would be reduced and so there would be no doubts about the efficacy of the method. Patients with less severe degrees of cellulite only lose the excess related to cellulite.

 As obesity and edema are deposit diseases, patients with these two conditions were excluded: the objective of this study was to evaluate cellulite. However, cellulite, obesity, and edema may be present in the same patient given the synergy of the three conditions thereby increasing the cellulite. This we denominate as the cellulite complex. Slimming or reducing the edema in isolation improves this cellulite complex; however, this is not the correct way to treat cellulite. Thus, an accurate diagnosis and specific treatment for each disease is crucial; a mistake that repeatedly occurs in the evaluation of these patients.

 The technique employed in this work aims at physiologically stimulating the lymphatic system; this difference, compared to other therapies, allows the results to be maintained for years. To achieve reductions in size is simple but increases are normally seen soon after ceasing treatment.

This intensive method revolutionizes the treatment of cellulite. The technique was conceived after developing new procedures to improve lymphedema. Patients under treatment for lymphedema observed that the cellulite was also improving. From these observations and a review of the literature about the subject, we came up with a physiopathological hypothesis about this condition. Our hypothesis is that lymphatic stasis results in an accumulation of all the substances drained by the lymphatic system [17]. In the initial phase of cellulite, an accumulation of fluids and other substances is identified in the interstitial space. Progression to the clinical condition is related to the concentration of these substances associated with local reactions due to this buildup. Thus, cellulite is a disease caused by accumulation. The hypothesis explains that the main cause of lymphostasis is related to the action of female hormones on the contraction of the lymphatics and the tonus of the vessels as well as exacerbation of substances in the interstitial space.

 The therapeutic proposal is different to the majority of the published reports as it approaches the condition of restoring the pathophysiology involved in alterations to the interstitial space. It does not cure cellulite when there are triggering stimuli, though improvement of the physiology allows a balance of the hemostasis of the interstitial space and control of the cellulite. The important thing is not to injure the subcutaneous tissues during treatment as this may further aggravate the cellulite. Many methods aim at destroying the adipose cells and surrounding tissue, which can result in reduced volume by an inflammatory mechanism with lesions of the vessels thereby causing flaccidity as is observed with many of these treatments.

 Another aspect to be considered is that this form of treatment does not lead to any weight loss. One hypothesis that explains these great reductions in size without weight loss is redistribution throughout the entire organism. Thus it is possible to have reductions in the size of the abdomen and thighs of more than 10 cm without a reduction in weight. One patient treated by this method lost 7 cm at the greatest measurement point of the thigh and put on two kilograms. This surprised her friends who thought she had lost weight and not gained weight. According to this hypothesis, the main substance mobilized is hyaluronic acid. This is compatible with the experience of patients with very dry skin who report a great improvement after treatment. Hence, this technique promises to revolutionize the treatment of cellulite.

A series of investigations are being carried out by the authors aiming at providing more data; one book is already available with further information about the different aspects of this approach [11]. We have been researching this technique for 10 years and have observed that several factors interfere in the results, and so the correct use of the method is crucial. Currently, we have more than 500 patients evaluated using different forms of this method and of the results and failings that it presented and how the difficulties were solved. The difficulties found in relation to the evaluation of these patients are related to a reliable method of evaluation before and after treatment. In more severe patients, the reductions can reach 10 cm in 10 days without weight loss, and there is no doubt in respect to the results. However, mild degrees, with variations of 1 to 2 cm, are more difficult to evaluate. Thus, a cheap method that provides more precise measurements is necessary to help to evaluate these patients.

## 5. Conclusions

 The form of intensive treatment of cellulite proposed in this study allows significant reductions of this disease.

## Figures and Tables

**Table 1:** Measurements 5, 10, and 15 cm above the gluteal fold before and after treatment.

Patient no.	*N*	Before treatment (cm)	After treatment (cm)	Difference (cm)
1	5 cm	94	90	4.0
	10 cm	92	88	4.0
	15 cm	88	86	2.0

2	5 cm	103	101	2.0
	10 cm	99.8	96	2.8
	15 cm	93	90.2	2.8

3	5 cm	111.2	104	7.2
	10 cm	110.9	102	10.5
	15 cm	105.6	99	6.6

4	5 cm	100	97	3.0
	10 cm	100.6	95.8	4.8
	15 cm	97.5	95	2.5

5	5 cm	107	103.3	3.7
	10 cm	106	100	6.0
	15 cm	103.5	98	5.5

6	5 cm	107.8	103	4.8
	10 cm	106.6	102	4.4
	15 cm	104	98	6.0

7	5 cm	108	98.5	9.5
	10 cm	103	93.5	9.5
	15 cm	96	90	6.0

8	5 cm	106	100	6.0
	10 cm	105	98.2	6.8
	15 cm	103	95	8.0

9	5 cm	106.5	100	6.5
	10 cm	104	98	6.0
	15 cm	103	98	5.0

10	5 cm	110	98.3	11.7
	10 cm	108	96	8.0
	15 cm	104	101	3.0

Mean		102.9	97.2	5.6*

*P  value < 0.0001 (paired *t*-test).

**Table 2:** Measurements of the right thigh 5 and 10 cm below the gluteal fold before and after treatment.

Patient no.	*N*	Before treatment (cm)	After treatment (cm)	Difference (cm)
1	5 cm	53	49	4.0
	10 cm	47.5	45	2.5

2	5 cm	58	55	3.0
	10 cm	51	49	2.0

3	5 cm	62.5	57	5.5
	10 cm	58.2	51.2	7.0

4	5 cm	48.3	45	3.3
	10 cm	56.5	53	3.5

5	5 cm	54	50	4.0
	10 cm	64	61	3.0

6	5 cm	62	58	4.0
	10 cm	61.5	56	5.5

7	5 cm	59.3	51	8.3
	10 cm	53	48	5.0

8	5 cm	49	47	2
	10 cm	52	50	2.0

9	5 cm	64	54.5	9.5
	10 cm	62	52	10

10	5 cm	65	58	7.0
	10 cm	66	60	6.0

Mean		57.3	52.5	4.9*

*P  value < 0.0001 (paired *t*-test).

**Table 3:** Measurements of the left thigh 5 and 10 cm below the gluteal fold before and after treatment.

Patient #	N	Before treatment (cm)	After treatment (cm)	Difference (cm)
1	5 cm	53	49	4.0
	10 cm	48	45	3.0

2	5 cm	58	53	5.0
	10 cm	51	49	2.0

3	5 cm	62.5	57	5.2
	10 cm	58.5	51.2	7.3

4	5 cm	48	45	3.0
	10 cm	56.5	53.6	3.1

5	5 cm	54.5	50.6	4.1
	10 cm	64	61	3.0

6	5 cm	63.5	57.5	6.0
	10 cm	60	58	2.0

7	5 cm	59.5	51	8.5
	10 cm	53	48	5.0

8	5 cm	49	47	2.0
	10 cm	52	50	2.0

9	5 cm	64	54	10
	10 cm	62	52.3	4.3

10	5 cm	65.5	58	7.5
	10 cm	66	60	6.0

Mean		57.4	49.9	4.7*

*P  value < 0.0001 (paired *t*-test).

**Table 4:** Measurements before and after treatment at the gluteal fold region.

Patient #	Right leg		Left leg	
	Before treatment (cm)	After treatment (cm)	Before treatment (cm)	After treatment (cm)
1	57	55	57	55
2	59.5	55	59.3	55
3	64	60	64	60.2
4	57	53	57	53
5	64	61	64	61
6	63	60	63	60
7	58	56.2	59.5	56
8	58.5	54	58	54
9	62.5	56	62	56
10	68.5	60	68	60

Mean	*61.2	**57.0	*61.2	**57.0

*P  value < 0.0001 (paired *t*-test). **P  value < 0.0001 (paired *t*-test) *before **after result.

**Table 5:** Measurements at the navel before and after treatment.

Patient #	Before treatment (cm)	After treatment (cm)	Difference (cm)
1	78.9	77	1.9
2	74	72	2.0
3	87.3	84	3.3
4	85.5	82.5	3.0
5	86.5	80	6.5
6	94.6	90	4.6
7	84	78	6
8	90	87	3
9	95.5	88.7	6.8
10	90.8	85.5	2.3

Mean	86.7	82.5	3.94*

*P  value < 0.0001 (paired *t*-test).

**Table 6:** Measurements before and after treatment 5 cm above the navel.

Patient #	Before treatment (cm)	After treatment (cm)	Difference (cm)
1	72.5	68	4.5
2	66.6	62	4.6
3	96.3	92	4.3
4	77	76	1.0
5	79.5	76	3.5
6	96	90	6
7	66	61	5
8	78.5	75	3.5
9	91	89	2
10	84	78	6

Mean	80.7	76.7	4.0*

*P  value < 0.0001 (paired *t*-test).
